# Strong Magneto-Optical Kerr Effects in Ni-Doped ZnO Nanolaminate Structures Obtained by Atomic Layer Deposition

**DOI:** 10.3390/ma16196547

**Published:** 2023-10-04

**Authors:** Armando Galluzzi, Krastyo Buchkov, Blagoy S. Blagoev, Albena Paskaleva, Ivalina Avramova, Vladimir Mehandhziev, Peter Tzvetkov, Penka Terziyska, Daniela Kovacheva, Massimiliano Polichetti

**Affiliations:** 1 Department of Physics ‘E.R. Caianiello’, University of Salerno, via Giovanni Paolo II, 132, Fisciano, I-84084 Salerno, Italy; agalluzzi@unisa.it (A.G.); mpolichetti@unisa.it (M.P.); 2 CNR-SPIN Salerno, via Giovanni Paolo II, 132, Fisciano, I-84084 Salerno, Italy; 3 Institute of Solid State Physics, Bulgarian Academy of Sciences, 72 Tzarigradsko Chaussee Blvd., 1784 Sofia, Bulgaria; blago_sb@yahoo.com (B.S.B.); paskaleva@issp.bas.bg (A.P.); vlado_bm@yahoo.com (V.M.); penka@issp.bas.bg (P.T.); 4 Institute of General and Inorganic Chemistry, Bulgarian Academy of Sciences, Acad. G. Bonchev Street, bl. 10, 1113 Sofia, Bulgaria; iva@svr.igic.bas.bg (I.A.); tzvetkov@svr.igic.bas.bg (P.T.); didka@svr.igic.bas.bg (D.K.)

**Keywords:** ZnO, atomic layer deposition, magneto-optical Kerr effect

## Abstract

The magneto-optical (MO) Kerr effects for ZnO and ZnO:Ni-doped nanolaminate structures prepared using atomic layer deposition (ALD) have been investigated. The chemical composition and corresponding structural and morphological properties were studied using XRD and XPS and compared for both nanostructures. The 2D array gradient maps of microscale variations of the Kerr angle polarization rotation were acquired by means of MO Kerr microscopy. The obtained data revealed complex behavior and broad statistical dispersion and showed distinct qualitative and quantitative differences between the undoped ZnO and ZnO:Ni-doped nanolaminates. The detected magneto-optical response is extensively inhomogeneous in ZnO:Ni films, and a giant Kerr polarization rotation angle reaching up to ~2° was established. This marks the prospects for further development of magneto-optical effects in ALD ZnO modified by transition metal oxide nanostructures.

## 1. Introduction

The broad spectrum of physical properties of and practical perspectives on diluted magnetic semiconductors (DMS) is one of the main research topics both for fundamental science and technology. The prospects for unified control over the electronic nature and charge carrier dynamics in the semiconductor with the robustness and coherence of the ordered magnetic spin state are constantly being developed, especially in the frames of spintronics and micro(nano)electronics [1,2,3]. The zinc oxide (ZnO), a prominent semiconducting material [4], is extensively explored for the possibilities to create and control the magnetic state altogether with a diverse set of physical degrees of freedom—wide direct band gap of 3.37 eV, moderate electrical conductivity and optical transparency. In the “magnetism” context, the otherwise non-magnetic ZnO is one of the most investigated materials with a constantly expanding complexity. ZnO can be chemically modified with (3d) ferromagnetic transition metal (TM) elements such as Ni, Co, Fe, Mn, etc., which induce a room temperature ferromagnetic state; however, despite the high investigation activity on this material, many aspects are still unresolved.

Multifold Ni-doping effects in ZnO are widely studied in different structures such as thin films [5] and heterostructures [6] as well as in nano-sized objects with particular morphology and geometry such as nano-rods, wires, tubes, spheres, etc. [7,8]. Both metallic elements (Zn and Ni) have comparable ionic radii (Ni^2+^ = 0.55 Å and Zn^2+^ = 0.6 Å), which permits the effective substitution and solubility of Ni in the main ZnO matrix without a detrimental impact on the crystal structure and composition [9]. The chemically modified Zn(Ni)O composite nanostructures show an altered bandgap electronic structure with a significant effect on the physical properties. The most recent studies are focused on the following: (i) the main optical properties (absorbance, photoluminescence) and constants, photoconductivity and low optical losses, linear and non-linear (third order) optical susceptibilities with opto-electronic prospects in quantum technologies [10], photo-detection [11] and solar cells with high detector responsivity; (ii) the development of enhanced catalyst function [12,13] and surface (gas species) adsorption in numerous green chemistry applications, especially for photo-chemical [14] and gas sensing applications [15]; and (iii) notable thermoelectric [16] and acoustoelectronic [15] (with larger insertion loss and phase shift) characteristics. 

For transition metal oxide TMO-doped ZnO, it is challenging to identify the origin (intrinsic or mediated by secondary phases) and to analyze the phenomenology of magnetism as it is strongly dependent on the deposition approach and processing conditions of materials. In the case of Zn(Ni)O thin films, the established effects include room temperature ferromagnetism, super-paramagnetism [17], strong magnetic anisotropy [18], and conventional paramagnetic behavior—determined especially by the doping level as well as the preparation technique [19]. 

The magneto-optical (MO) properties of ZnO-based DMS are also investigated in order to combine, in a practical direction, the notable optical transparency together with magnetism. Advanced (spectroscopic) techniques such as magnetic–circular dichroism [20,21], MO Faraday [22,23], and the MO Kerr effect [24,25] (MOKE) provide the possibility to explore, in detail, the electronic band structure and origin of magnetism at the unit cell level. The MOKE technique explores the linear polarization effect of the light reflected from the magnetic surface directly in relation to the microscale magnetic hysteresis. Depending on the magnetization vector orientation with respect to the thin film surface and to the plane of incident light, the MO Kerr effect diverges in three different configurations: longitudinal, polar and transverse. In addition, the technique is sensitive to the imbalances of the electronic spin-state nature, especially for nanostructured (ultrathin) samples even within the 2D limit [26]. The (linearly) polarized incident light reflected (with a change in the phase and intensity) from the magnetized medium becomes ellipsoidally polarized with the rotation of the polarization plane, marked by the Kerr angle $\theta_{Kerr}$. Expectedly, the Kerr parameters are also influenced by any simultaneous modification of the electronic states under additional fields (electric, thermal, structural, and interface) giving rise to the implementation of the effects in a plethora of device and sensor applications.

Consequently, the development of the MOKE technique is progressing in various research and practical directions [27]. MO Kerr microscopy is an irreplaceable tool for studying and visualizing the spin system dynamics, magnetic domain textures and quasi-particles (such as skyrmions). Some of the state-of-the-art developments are related to the novel antiferromagnetic spintronics, magneto-plasmonics, and 2D and topological materials and are especially focused on the improvement of spatial, temporal, and spectral resolution [28,29,30,31,32]. In addition, the recent advancements in the field of topological insulators can give new impulse for a revival of MOKE-based memory drive technology [33]. 

ZnO thin films (including their doped counterparts) with various structural and physical properties can be prepared using a number of deposition techniques, e.g., DC/RF magnetron (cathode) sputtering [34], pulsed laser deposition [35], chemical (physical) vapor deposition [36], etc. Atomic layer deposition (ALD) is an advanced deposition technique for the preparation of high-quality films with precise thickness control on an atomic level, high film uniformity on large surfaces, and a low number of defects. ALD allows the preparation of films on organic substrates with low deposition temperatures and uniform covering of complex 3D high-aspect-ratio substrates, etc. All of these advantages are due to the specific nature of the ALD process in which alternating sequential pulses of gas precursors are introduced into the chamber and react with the surface of the substrate in a self-limiting manner. 

In this context, ALD enables nanostructure engineering [37] of ZnO with suitable TMO compounds. There have been extensive investigations for establishing the correlation between the physical properties of ALD ZnO thin films and the deposition process variables [37,38,39] and their optimization towards specific application requirements. This is especially valid for the research trends to unite the semiconducting (ZnO) and magnetic-Ni-based TMO. 

In this work, we present magneto-optical characterization of the spatial magnetization of ALD ZnO nanolaminates (deposited on p-Si (100)) by means of scanning Kerr microscopy. The comparative MOKE analysis of the undoped ZnO and Ni-doped ALD nanolaminate structures was performed by scanning the 2D gradient maps of the local variation of the Kerr rotation angle. The samples were prepared using the advanced nano-engineering capabilities of the ALD. 

It should be mentioned that this research trend is rarely explored both in terms of the deposition approach and magneto-optical analysis and will provide future guidelines for the improvement of film architecture and the implementation of strong MO Kerr effect properties.

## 2. Experimental Details

### 2.1. The Atomic Layer Deposition Process: Nanolaminate ALD Protocol 

**Figure 1:**
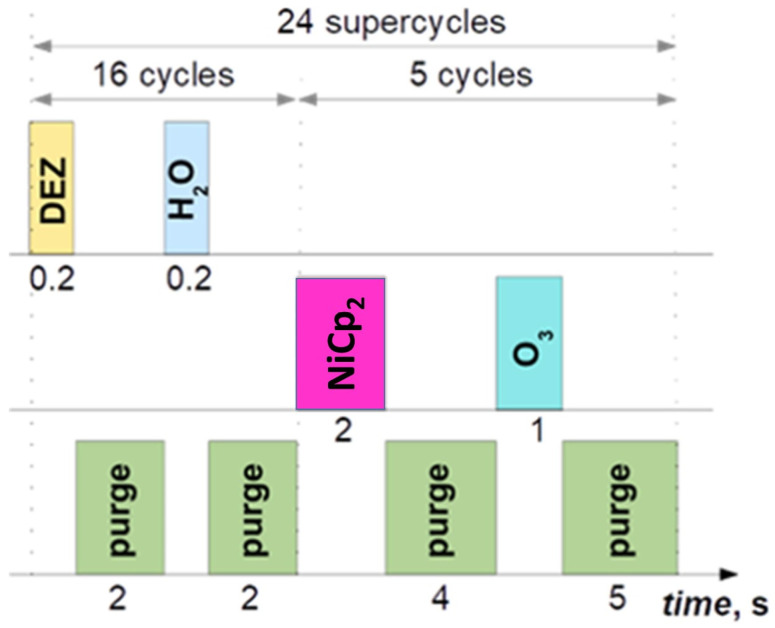
ALD ZnO deposition: Ni protocol.The ALD precursor pulse and purging (p) duration for subcycles were as follows: DEZ/p/DI H_2_O/p = 0.2/2/0.2/2 s and NiCp_2_/p/O_3_/p = 2/4/1/5 s, respectively. To enhance the sublimation process of the nickelocene precursor, its vapor pressure was increased by heating the precursor container up to 80 °C.

The undoped ZnO and Ni-doped ZnO nano-films were grown on p-Si (100) via the standard thermal ALD process (Figure 1) using a Beneq TFS-200 reactor system. The ALD system is equipped with an HS-300 hot source container filled with NiCp_2_ (nickelocene) powder as a volatile solid Ni precursor. In addition, Diethylzinc (DEZ) (Strem Chemicals, Inc. Newburyport, MA, USA) as a Zn precursor and deionized (DI) H_2_O as an oxidant to obtain ZnO matrix were used. To obtain a pure ZnO film, DEZ/DI H_2_O cycle was repeated 1000 times and the reactor chamber was heated up to 165 °C. For the doped ZnO structure (ZnO:Ni), 16 cycles of DEZ/DI H_2_O were followed by 5 cycles of NiCp_2_/O_3_ in 1 supercycle at 180 °C, where the supercycle repetitions were 24 times. A continuous N_2_ flow at 300 sccm (standard cubic centimeters per minute) as carrier and purging gas was used during the ALD process. 

### 2.2. Magneto-Optical Kerr Magnetometry

The magneto-optical investigations were performed at ambient temperature using a Quantum Design—Durham (Cambridge, UK) Magneto Optics: NanoMOKE-3^@^ Magneto Optical Kerr magnetometer under longitudinal field geometry. The maximum alternating field amplitude reached ~4000 Oe at a frequency of ~2.1 Hz. Scanning laser microscopy (SLM), which was used for performing the experiments, is capable of magnetic domain imaging with investigation of the local variation in magneto-optical Kerr rotation angle. In SLM-based imaging, a 660 nm semiconductor laser is deflected by a high-precision computer-controlled system of galvanometric mirrors. This allows the selection of different points on the surface of the sample and to analyze local magnetic response. By measuring and mediating many Kerr hysteresis loops for each selected point, color gradient maps of the Kerr rotation angle ($\theta_{Kerr}$) at hysteresis saturation have been constructed. By selecting an array of locations on the surface, a 2D mapping of the material Kerr magnetization properties can be obtained. In our case, arrays (X µm × Y µm) with various areas were investigated, with a magnetic pixel resolution ~10 µm. However, due to the specific strong magnetization response of the ZnO:Ni nanolaminate, we have improved the scan resolution to 5 µm sized cluster over different sectors. 

### 2.3. XPS, XRD and Spectroscopic Ellipsometry Experimental Details

The XPS analyses were performed on a Kratos AXIS Supra (Kratos Analytical Ltd., Manchester, UK) spectrometer with an Mg X-ray source under a vacuum level better than 10^−8^ Pa at a 90° take-off angle. Each analysis consisted of a standard survey scan from 0 to 1200 eV and a pass energy of 160 eV with a 0.5 eV step. For the high-resolution analysis, the number of sweeps was increased and the pass energy was lowered to 20 eV at steps of 100 meV. The C_1s_ photoelectron line at 285 eV was used for calibration of recorded spectra.

The ellipsometry measurements were performed using a Woollam@ M2000D (Lincoln, NE, USA) rotating compensator spectroscopic ellipsometer in a 193 to 1000 nm wavelength range. The thicknesses of the ZnO layers of each sample were determined by modeling the experimental ellipsometry parametric functions Ψ (amplitude ratio) and Δ (phase difference). The fit procedure is described elsewhere [40]. The experimental data were acquired at angles of incidence of 45, 65, 70 and 75 degrees. The samples were represented by a silicon substrate with three layers on top of it: a 2.59 nm-thick native silicon oxide, a ZnO layer, and a roughness layer. The roughness layer was simulated by 50% voids and 50% ZnO. The silicon substrate and the native silicon oxide were modeled using the optical constants from the instrumental software CompleteEASE^@^ v.5.19. The ZnO layer was represented by the general oscillator model. The thickness of the ZnO:Ni was determined to be 75.8 nm, and its surface roughness was 4.9 nm. The thickness of the undoped ZnO layer was determined to be 226.1 nm, and its surface roughness was 13 nm. 

The X-ray diffraction (XRD) patterns were collected within the 2θ range from 10° to 80° with a constant step of 0.02° on a Bruker (Bruker AXS, Karlsruhe, Germany) D8 Advance diffractometer with CuKα radiation and a “Lynx Eye” detector at room temperature. The phase identification was performed with the DiffracPlus EVA v.4.0 program using the ICDD-PDF2 Database (2021). The unit cell parameters and mean coherent domain size were determined with the whole powder pattern fitting procedure (Pawley fit) using the Bruker Topas v.4.2 program.

## 3. Results, Analysis and Discussion

### 3.1. Structural Characterization via XRD Analysis

**Figure 2:**
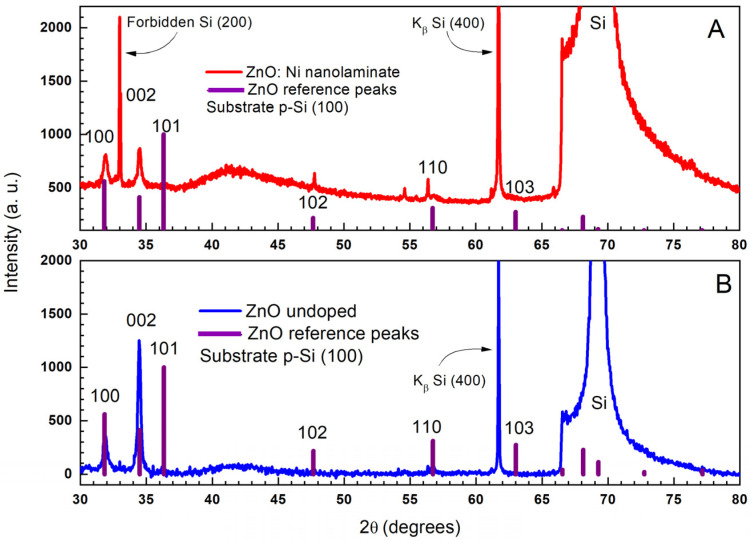
XRD comparison analysis of ZnO (**A**) and ZnO:Ni. (**B**) ALD nanolaminates with the positions and miller indices of the corresponding ZnO reference peaks. Some of the presented data [41] are reprinted with permission of IOP under CC BY 3.0 license.

The structural and morphological features of undoped ZnO layers as well as those caused by doping effects in ZnO:Ni were analyzed using XRD analysis. The diffraction patterns for both samples are presented in Figure 2. There is a match with a typical (for ZnO) diffraction pattern of wurtzite-type hexagonal structure and nanoscale crystallites (as seen by the broadening of the diffraction peaks). The average coherent scattering domain size determined by diffraction measurements is 34.6 (8) nm for ZnO and 28.4 (12) nm for ZnO:Ni. 

Detailed comparison with the reference diffraction pattern from a powder sample with isometric dimensions showed that for the undoped ZnO layer, the hexagonal planar crystallites are oriented in a way that the (002) plane is perpendicular to the substrate plane and the c-axis is parallel to the conventional growth direction of ALD layer. In other words, there is a preferential orientation along the <002> direction. 

For Ni-doped ZnO, however, the predominant orientation is partially reoriented along the <100> direction. The unit cell parameters were determined as follows for ZnO—a = 3.244(1) Å, c = 5.208(1) Å; and for ZnO:Ni—a = 3.238(1) Å, c = 5.202(2) Å. This observation means that Ni ions are successfully incorporated into the ZnO structure, leading to a slight decrease in the lattice parameters. This result is expected since Ni^2+^ ions (IR = 0.55 Å) have a smaller radius than those of Zn^2+^ ions (IR = 0.6 Å). Additional analysis of the phase composition did not reveal any measurable traces of impurity Ni-based oxide phases within the sensitivity of the method.

### 3.2. Chemical Composition: XPS Analysis 

**Figure 3:**
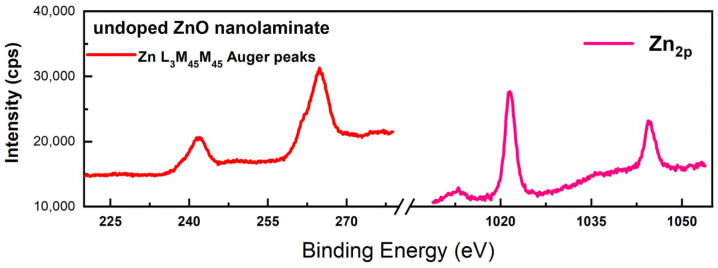
XPS spectrum for Zn2p and Zn L_3_M_45_M_45_ Auger peak for the undoped ZnO ALD sample.

**Figure 4:**
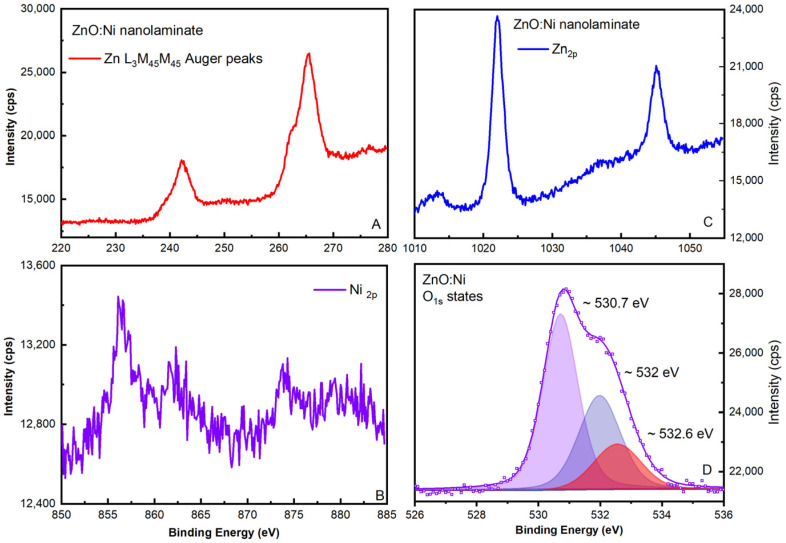
ZnO:Ni nanolaminate XPS spectral data for Zn L_3_M_45_M_45_ Auger peak for (**A**), peak traces for Ni 2p states (**B**) and Zn 2p (**C**). Peak deconvolution for O_1s_ state for identification of oxygen vacancy types and concentration (**D**). Some of the presented data [41] are reprinted with permission of IOP under CC BY 3.0 license.

The chemical composition analysis (valence and oxidation states with corresponding binding energies) of ZnO and ZnO:Ni nanolaminate samples was investigated using XPS. The spectral data for the valence state related to O_1s_ (oxygen vacancies), Zn_2p_, ZnLMM (Auger lines), and Ni^2+^ (doping traces) are presented in Figure 3 and Figure 4, correspondingly for both samples. 

The typical photoelectron spectrum for ZnO (to verify the successful formation) related to the presence the Zn_2p_ spectral line was identified. However, the further determination of the exact Zn chemical (valence) state is difficult since there is a minimal chemical shift in the associated Zn_2p3/2_ peak, which is close to the experimental sensitivity of the instrument regarding the binding energy resolution. Alternatively, the presence of the L_3_M_45_M_45_ Auger line provides additional information about the ZnO stoichiometry [42]. For ZnO samples, the Auger parameter was calculated by taking the binding energy of the Zn 2p_3/2_ photoelectron peak and kinetic energy of the Zn L_3_M_45_M_45_ Auger peak. This result additionally confirms the successful growth of ZnO during the ALD process in a correlation with the reference standards in the literature data [43]. Minimal detection traces related to the Ni doping are detected for ZnO:Ni nanolaminate. 

The acquired spectral data for the binding energy and shape of the Ni_2p_ photoelectron line is typical for hydroxide, possibly Ni(OH)_2_ [42]. This implies the successful incorporation of Ni complexes in the structure; however, it also still presumes a minimal doping level. The corresponding stoichiometric percentages for ZnO:Ni are estimated as follows: Zn~21.71% at., Ni~5.02, % at., and O~73.27% at. The O_1s_ photoelectron spectra were de-convoluted in order to determine the percentage of various oxygen groups (at the nanolaminate surface). 

The main oxygen component at 530.7 eV corresponds to oxygen bonded to Zn atom in the ZnO lattice. The second (~532 eV) and third (~532.5 eV) peaks are directly related to the oxygen vacancies and to adsorbed oxygen, OH^−^ groups or dissociated oxygen, respectively, on the surface of the oxide films.

The relative intensity of the second component is directly proportional to the concentration of oxygen vacancies [43] and allows more quantified comparison of the relative oxygen vacancy percentage (from the total oxygen content) within the main ZnO matrix for the studied undoped ZnO (17.19%) ([44] Supplementary) and ZnO:Ni (32.10%) nanolaminates. 

### 3.3. Magneto-Optical Kerr Effect Microscopy and Statistics

The longitudinal magneto-optical Kerr effect was studied and the data for undoped and Ni-doped ZnO ALD nanostructures were systematized in a comparative and statistical overview. The films were scanned in several different sectors, randomly taken on the entire sample surface to check the macroscopic homogeneity. Both samples show inhomogeneous clustered distribution with a broad spatial variation on a microscopic scale and, expectedly, due to TMO modification, the average magneto-optical response of the ZnO:Ni-doped nanolaminate is far more intense compared to the undoped ZnO. 

**Figure 5:**
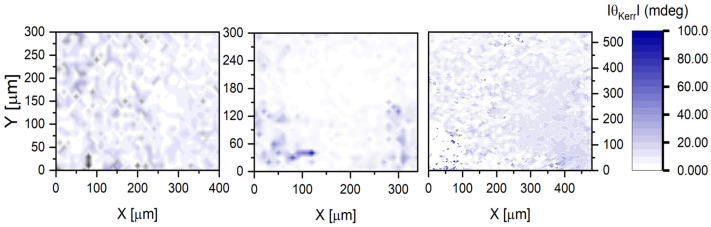
Maps of the Kerr rotation angle at saturation in three different sectors analyzed for the undoped ZnO nanolaminate.

**Figure 6:**
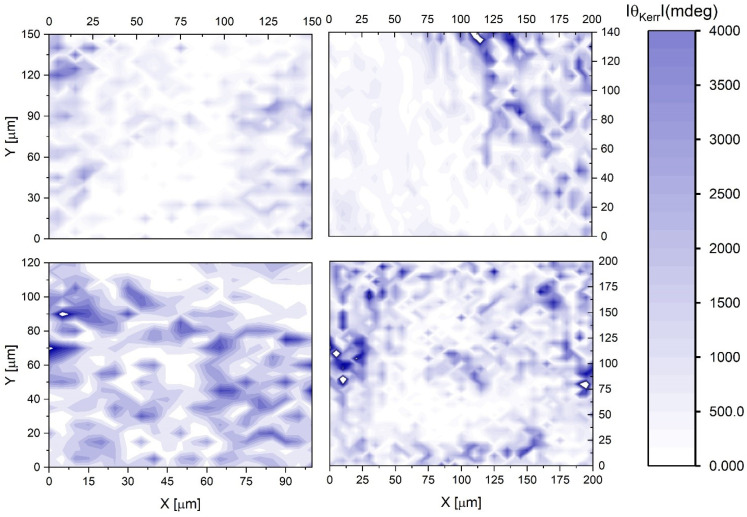
Two-dimensional maps of the Kerr rotation at saturation for ZnO:Ni nanolaminate scanned in four different sectors of the sample.

The sectoral mapping of the absolute value of the MO Kerr rotation angle $\left|\theta_{Kerr}\right|$ at MOKE hysteresis saturation is presented in Figure 5 and Figure 6 for both samples. 

For the undoped ZnO (Figure 5), the observed values of the Kerr angle modulus fall within the interval of 0 ÷ 50 mdeg. The gradient distribution maps are relatively homogenous.

The Kerr angle rotation maps for the ZnO:Ni-doped nanolaminate sample (Figure 6) show extreme magneto-optical responses with a more than two orders of magnitude higher with maximal $\left|\theta_{Kerr}\right|$ values reaching ~2° with extensive inhomogeneous spatial distribution. 

**Figure 7:**
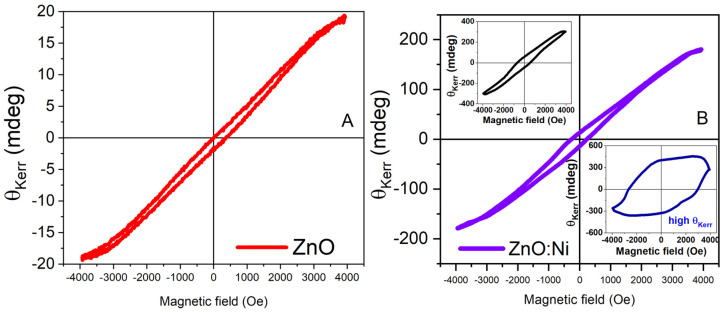
Selected *θ_Kerr_*(*H*) hysteresis loops for (**A**) undoped ZnO and (**B**) Ni-doped ZnO layers. The inset graphs show example curves with large hysteresis contours and Kerr angle values.

**Figure 8:**
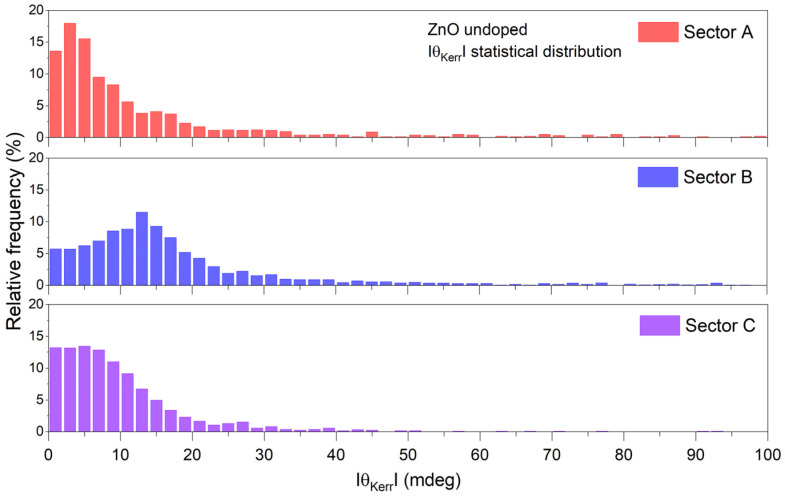
Statistical distribution of the Kerr angle rotation signal in three different sectors for undoped ZnO.

**Figure 9:**
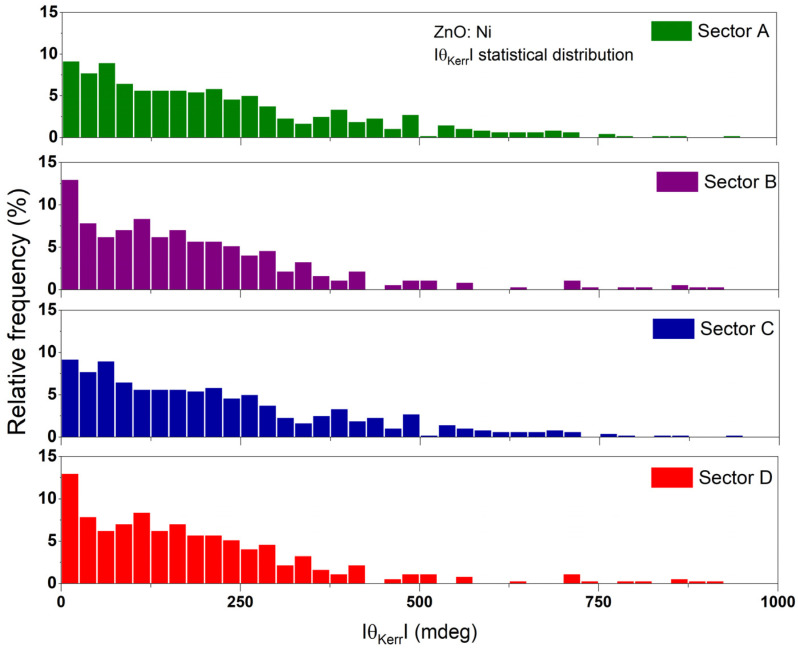
Statistical distribution of the Kerr angle rotation signal in four different sectors for the ZnO:Ni nanolaminate.

The detected non-uniformity of the magneto-optical behavior is correlated with the variable and aberrant micro-MOKE hysteresis. Typical $\theta_{Kerr}(H)$ contours for the un-doped and Ni-doped ZnO layers are presented in Figure 7. The observed variations are significant especially for the doped layers, where $\left|\theta_{Kerr}\right|$ can also reach significant values at the saturation magnetic field. In an effort to differentiate qualitative and quantitatively using the magnetic properties in the two kinds of samples, the histogram dispersion ($\left|\theta_{Kerr}\right|$ vs. values percentage) is shown in Figure 8 and Figure 9. For the undoped ZnO sample (Figure 8), the average value is ~0.02°. The majority of the data fall within the 0–0.05° range with a bin interval of 2 mdeg/0.002°.

The statistical distribution of $\left|\theta_{Kerr}\right|$ for ZnO:Ni-doped nanolaminate (Figure 9) is qualitatively and quantitatively different compared to its undoped counterpart. As already mentioned, the maximal $\left|\theta_{Kerr}\right|$ reached ~2°, with an average value of 0.494°, and 90% of all data are within 1° with a bin interval of 0.025°. Therefore, the ZnO:Ni-doped nanolaminate shows the very strong susceptibility of the magneto-optical response to the Ni doping and implies the randomized diffusion in the crystal structure. 

Enhanced polar Kerr rotation reaching a maximal value of $\theta_{Kerr}\;\sim \;0.7{}^{\circ}$ was also observed in different ZnO TMO-doped heterostructures under various architectures and with strong spectral and doping level dependences [24]. 

The prospects of the possible magnetism implication with soft magnetic behavior under an applied magnetic field in undoped ZnO is usually analyzed within the framework of the defect-induced d_0_ mechanism [45]. The net spin polarization is mediated by interrelated structural and stoichiometric deviations such as anion (oxygen VO) or cation (Zink VZn) vacancies as well as surface and interface/substrate effects [31,32,33]. 

The complexity of magnetic effects expands even more when room temperature ferromagnetism [2,46] is achieved via TM magnetic doping effects, as in the case of ZnO:Ni with further alteration of the chemical phase composition and possible foreign magnetic clusters embedded in the main ZnO matrix. Very generally, the phenomenology of ZnO magnetism mechanisms can be separated into two groups: (i) carrier-mediated exchange interactions between the localized magnetic moments, for instance, Rudermann–Kittel–Kasuya–Yosida (RKKY) [47] or Zener double exchange; or, (ii) alternatively, the creation of spin correlated structure can be related to the electrons trapped by the nanoscale structural defects (vacancies and/or secondary phases) and leads to orbital d-shell overlapping of the neighboring TM doping atoms with the formation of bound magnetic polarons (BMP) [48]. The fact that, due to the Ni doping, an overall strong magneto-optical behavior is observed, can be explained by the simultaneous influences of various (substitutional) effects on Zn/Ni position sites, variations in oxygen vacancy concentrations, and inclusions due to the interface effects in the ZnO:Ni layered matrix, as also revealed by the XPS elemental analysis. 

From another point of view, considering the nanolaminate architecture (with alternating ZnO layer and NiO layers), we can also note a prominent optical amplification (due to interference artefacts) of the MO Kerr rotation effect observed in complex transparent ZnO/TMO multilayered structures deposited onto highly reflective substrate material [49,50,51]. 

Following this general context, at the used laser wavelength (660 nm) and magnetic field range, the ALD ZnO:Ni reveals features of a high MO Kerr effect class material. Such a strong response marks the practical potential of the system. 

Several groups of materials with a diversity of physical natures show giant MOKE polarization rotation (comparison regarding polar Kerr effect); magnetic Fabri–Pérot heterostructure [52]; ferro-, ferri- and antiferromagnetic (multiferroics, garnets) systems ([53] and references therein); artificially nano-engineered metamaterials [54]; 2D materials [51]; nanostructured plasmonic dielectrics [55]; and topological insulators [56]. Generally, these are materials and systems that require more sophisticated process technology. Therefore, the presented results reveal that, by using a simple ALD scheme, it is possible to obtain a significant magneto-optical response in ZnO:Ni-doped nanolaminates.

## 4. Conclusions

In conclusion, we have presented initial comparative (structural, morphological, chemical composition and magneto-optical) studies of the MO Kerr effects of ALD prepared undoped ZnO and ZnO-Ni nanolaminate thin film structures. By means of MO Kerr scanning laser microscopy, the lateral change in magnetization was investigated. The acquired 2D maps and statistical histogram analysis revealed an inhomogeneous distribution (for both samples). The ZnO:Ni showed a notably strong magnetization response and a giant Kerr polarization rotation angle whose maximal values reached up to $\left|\theta_{Kerr}\right|\;\sim \;2{}^{\circ}$. 

Therefore, exploring the possibility to establish more precise and uniform magneto-optical control will require optimization of the atomic layer deposition process (including possible annealing steps) to improve the homogeneity of Ni diffusion in the ZnO matrix. 

## Data Availability

The datasets that support the findings in this study are available from the corresponding author upon reasonable request.
